# Hybrid Algorithm Based on Ant Colony Optimization and Simulated Annealing Applied to the Dynamic Traveling Salesman Problem

**DOI:** 10.3390/e22080884

**Published:** 2020-08-12

**Authors:** Petr Stodola, Karel Michenka, Jan Nohel, Marian Rybanský

**Affiliations:** 1 Department of Intelligence Support, University of Defence, Kounicova 65, 662 10 Brno, Czech Republic; karel.michenka@unob.cz (K.M.); jan.nohel@unob.cz (J.N.); 2 Department of Military Geography and Meteorology, University of Defence, Kounicova 65, 662 10 Brno, Czech Republic; marian.rybansky@unob.cz

**Keywords:** dynamic traveling salesman problem, combinatorial dynamic optimization problem, ant colony optimization, simulated annealing, hybridization, metaheuristic algorithm

## Abstract

The dynamic traveling salesman problem (DTSP) falls under the category of combinatorial dynamic optimization problems. The DTSP is composed of a primary TSP sub-problem and a series of TSP iterations; each iteration is created by changing the previous iteration. In this article, a novel hybrid metaheuristic algorithm is proposed for the DTSP. This algorithm combines two metaheuristic principles, specifically ant colony optimization (ACO) and simulated annealing (SA). Moreover, the algorithm exploits knowledge about the dynamic changes by transferring the information gathered in previous iterations in the form of a pheromone matrix. The significance of the hybridization, as well as the use of knowledge about the dynamic environment, is examined and validated on benchmark instances including small, medium, and large DTSP problems. The results are compared to the four other state-of-the-art metaheuristic approaches with the conclusion that they are significantly outperformed by the proposed algorithm. Furthermore, the behavior of the algorithm is analyzed from various points of view (including, for example, convergence speed to local optimum, progress of population diversity during optimization, and time dependence and computational complexity).

## 1. Introduction

In recent years, both in civilian and military environments, considerable attention has been paid to optimizing tasks and problems in a dynamically changing environment. In the military environment, this attention is closely related to the growing increase in newly introduced technical means (e.g., unmanned systems), which are programmed to perform individual tasks by means of optimization techniques and algorithms. These techniques would make individual activities more efficient by finding a high-quality solution to the problem at hand.

The problem in which the input variables change in time is called the dynamic optimization problem (DOP). The optimization of DOPs aims to keep track of changes and adapt to them in order to effectively find high-quality solutions [1]. A simple example of a combinatorial DOP is the dynamic traveling salesman problem (DTSP). In this article, a metaheuristic approach for the DTSP is proposed and evaluated on a set of experiments.

This article is organized as follows. Later in this section, the mathematical formulation of the DTSP is presented and the authors’ motivation for dealing with this problem is discussed. Section 2 briefly reviews the literature connected with the heuristic approaches and algorithms of the DTSP. In Section 3, the new hybrid metaheuristic algorithm is proposed. Section 4 evaluates the algorithm on a set of benchmark instances and compares the results with other state-of-the-art metaheuristic techniques; this section also analyzes and discusses the parameters and behavior of the proposed algorithm. Finally, Section 5 concludes the article and offers several possibilities for future work.

### 1.1. Dynamic Traveling Salesman Problem

The DTSP is defined as a sequence of static TSP sub-problems (iterations). Let $I_{0}$ be the primary sub-problem which determines the number of vertices and their positions. The first iteration $I_{1}$. is derived from the primary sub-problem $I_{0}$; both the positions of the vertices and their number may be affected (the extent of change is generally not limited). Each successive iteration is derived from the previous one; $I_{i-1}\to I_{i}$ for i=1, …, I. where I is the total number of iterations (except the primary sub-problem).

Let $V=\left\{V^{0},V^{1},\ldots ,V^{I}\right\}$. be a set of all TSP sub-problems; $V^{i}\in V$ is a set of vertices representing an i-th TSP iteration, i.e., $V^{i}=\left\{V_{1}^{i},V_{2}^{i},\ldots ,V_{N^{i}}^{i}\right\}$ where $N^{i}$ is the number of vertices in this iteration. The cost of traveling between any pair of vertices in the i-th iteration is $c_{jk}^{i}=cost\left(V_{j}^{i},V_{k}^{i}\right)$; if the DSTP problem is symmetric, then $c_{jk}^{i}=c_{kj}^{i}$ for all $V^{i}\in V$. and $V_{j}^{i},V_{k}^{i}\in V^{i}$, otherwise, the problem is asymmetric.

A solution to the DTSP problem is given by a set of routes $R=\left\{R^{0},R^{1},\ldots ,R^{I}\right\}$, one for each iteration. A route in an iteration starts in an arbitrary initial vertex, visits all other vertices just once in some order, and then returns back to the initial vertex: $R^{i}=\left\{R_{1}^{i},R_{2}^{i},\ldots ,R_{N^{i}}^{i},R_{N^{i}+1}^{i}\right\}$ where $R_{1}^{i}=R_{N^{i}+1}^{i}$, $R_{j}^{i}\in V^{i}$ for all $j=1,\;2,\;\ldots ,\;N^{i}+1$, and $R_{j}^{i}\neq R_{k}^{i}$ for all $j,k=1,\;2,\;\ldots ,\;N^{i}$ and j≠k.

Quality C of each solution R of the DTSP can be calculated according to Formula (1) as a sum of costs of individual routes $C^{i}$ of all TSP iterations (i=0, 1, 2, …, I). The cost of each route is calculated as a sum of all distances traveled within this route (see Formula (2)). The function $\mathrm{cost}\left(R_{j}^{i},\;R_{j+1}^{i}\right)$ represents the distance between vertices $R_{j}^{i}$ and $R_{j+1}^{i}$ on route $R^{i}$.

(1) $$C=\overset{I}{\underset{i=0}{\sum }}C^{i}$$

(2) $$C^{i}=\overset{N^{i}}{\underset{j=1}{\sum }}\mathrm{cost}\left(R_{j}^{i},\;\;R_{j+1}^{i}\right).\;$$

The objective of the DTSP is to find such a route $R^{*}$ with a minimum value of $C^{*}$. This route $R^{*}$ is considered optimal when $C^{*}\leq C$ for all other feasible solutions in the state space. As the DTSP is composed of a series of independent TSP iterations, the optimal solution can be found as a set of optimal routes $R^{i*}$ for individual iterations ($C^{*}=C^{0*}+C^{1*}+\ldots +C^{I*}$). The aim of the algorithms proposed for the solution is expressed in Formula (3); this means to find the highest quality solution possible, i.e., with the lowest total cost possible (optimal in the best case).

(3) minimize(C)

### 1.2. Motivation

The military application of the DTSP can be illustrated using the unmanned ground vehicle (UGV) used in systems of Command, Control, Communication, Computer, Intelligence, Surveillance, and Reconnaissance (C4ISR). For example, as a consequence of the commander’s need to obtain information from the area of intelligence responsibility (AIR) via a UGV, the C4ISR system plans a route for this UGV starting from its base and returning after visiting all the waypoints in the AIR and thus obtaining the necessary information. Due to the need to get information as quickly as possible, the task is to optimize its time and plan the trip as efficiently as possible. A UGV using the DTSP, taking into account the distances between each programmed point in the designated area, can generate a route and start searching for information. However, due to unpredictable circumstances (enemy, terrain, weather, etc.), it is difficult to consider traffic restrictions or delays that may affect the planned route. Traffic restrictions may change the scheduled time due to conditions, and the UGV will need to find an alternative route quickly to avoid losing the time it takes to explore the specified space.

The Tactical Decision Support System (TDSS), as part of the C4ISR system, is being developed at the University of Defence, Czech Republic. The objective of this system is to assist the tactical commanders of the Army of the Czech Republic in their decision-making processes [2]. The DTSP solution proposed in this article is used in one of the models of military tactics implemented in the TDSS. The objective of this model is to plan the reconnaissance operation for the AIR using a UGV in a changing and uncertain environment. The model places a number of waypoints in the AIR so that as much area as possible is explored using a minimum number of waypoints. All the waypoints need to be visited just once and, after visiting the last waypoint, the UGV returns. If the environment changes, the positions of the waypoints may also change and a new route is required. The algorithm proposed in this article is used to plan the route of the UGV. More information about the model can be found in [3]; the general topic of decision support for commanders is explored in [4,5,6,7,8,9,10].

### 1.3. Contributions

The main contributions of this article are as follows:

- A novel hybrid metaheuristic algorithm using the unique combination of ant colony optimization (ACO) and simulated annealing (SA) principles was proposed for the dynamic traveling salesman problem.
- The algorithm is universally applicable for symmetric and asymmetric as well as metric and non-metric DTSP problems.
- The performance of the algorithm was evaluated on a set of benchmark instances including small, medium, and large DTSP problems.
- The results on the benchmark problems were evaluated using a set of four different experiments in order to show the significance of (a) the hybridization of both metaheuristic principles and (b) knowledge about the dynamic changes in successive iterations.
- The results were compared with four other state-of-the-art metaheuristic algorithms based on the ant colony optimization or discrete particle swarm optimization approach.
- A detailed analysis of the behavior of the algorithm was conducted, including the influence of parameters on the convergence speed, progress of population diversity during optimization, degree of improvement caused by simulated annealing, and time dependence and computational complexity analysis.
- A possibility for further improvement of the solution for the DTSP was discussed and assessed using the *k*-opt optimization principle.

## 2. Literature Review

The TSP problem was one of the first NP-hard problems to be studied; the research started at Princeton University in the 1930s [11]. Since that time, the problem has attracted many researchers and thousands of different approaches and algorithms have been developed. The first attempts to solve the problem used linear programming techniques; the objective is to minimize a linear function subject to specified linear equality/inequality constraints [12]. This approach has evolved and is still being used in many solutions. One of the most well-known and popular solutions is the Concorde TSP solver [13] that participated in finding the optimal solution for all benchmark instances from the TSPLIB [14], including the largest instance with 85,900 vertices. This instance was solved by Concorde in April 2006, consuming over 136 CPU years on clusters with 256 processors [15].

Exact methods are not always suitable for larger instances. Therefore, many heuristic and metaheuristic approaches have been proposed. One of the most successful heuristics was developed by Lin and Kernigham [16]. Although the authors limited their algorithms to problems with at most 110 vertices, they can successfully be applied to much larger instances [17]. The improved Lin–Kernigham algorithm called LKH was introduced by Helsgaun [18]; this algorithm provided the best solution reported thus far for the World TSP instance containing 1,904,711 vertices [19]. Different metaheuristic methods for the TSP also emerged using various principles such as ant colony optimization (ACO) [20], particle swarm optimization (PSO) [21], simulated annealing [22], genetic algorithms [23], and others [24], as well as their combinations [25,26,27].

All the algorithms and methods developed for the TSP can also be used for the DTSP; each iteration can be solved independently of the others. However, the knowledge that one iteration is not much different from the next can be used in favor of the algorithms with the benefit of achieving better solutions and/or shorter optimization time. The literature is, of course, less extensive than in the case of the TSP, but still, this topic has been covered by hundreds of publications. Therefore, only the newest research and publications concerning the DTSP are examined in this section.

The ACO principle was used in several cases for solving the DTSP. Mavrovouniotis and Yang [28] addressed the problem of population adaptation to the new environments in successive iterations by increasing the population diversity via transferring knowledge from previous environments to the pheromone trails using immigrant schemes; they proposed and implemented different immigrant schemes in their ACO algorithm, including random immigrants, elitism-based immigrants, and memory-based immigrants. Ma et al. [29] proposed an adaptive ACO algorithm; it consists of the evaluation of the degree of changes in successive iterations of the TSP, and then an adaptive pheromone initialization mechanism configured according to this degree is used. In their ACO solution, Chowdhury et al. [30] developed adaptive-large-neighborhood-search-(ALNS)-based immigrant schemes to transfer knowledge from the pheromone trails in one iteration to the next; they implemented their method in a real-life application for wildlife surveillance via drones. Some other publications proposing the ACO principle for the DTSP can be found in [31,32,33].

The discrete particle swarm optimization approach was adapted for the DTSP by Strak et al. [34]. The authors proposed their PSO algorithm with heterogeneous (non-uniform) parameter values; the parameters are set automatically for the critical PSO parameters based on discrete probability distributions. This approach proved to be more successful compared to the original PSO with homogeneous (uniform) parameter values [35]. Genetic algorithms are also used in several cases for the DTSP such as when [36] presenting an algorithm called the extended virtual loser genetic algorithm (eVLGA) or [37] presenting a genetic algorithm that feeds on Newton’s motion equation to show how route optimization can be improved when targets are constantly moving.

So far, there have not been many attempts to hybridize two heuristics together for the DTSP. One of the few can be found in the paper by Boryczka and Strak [38] where the authors connected discrete PSO with ACO principles. The proposed solution uses the virtual pheromone matrix which serves as a communication topology and provides information about the landscape of global discrete space. Mavrovouniotis, Muller, and Yang [39] integrated the memetic ACO algorithm with local search operators to improve solutions in the population. They apply these local search operators to the best solution found in the population in order to possibly improve this solution; a similar idea is used in this article, but instead of local search operators, simulated annealing (SA) is used.

The idea of hybridization of the ACO and SA have been already applied in some studies dealing with a variety of problems. For example, the combination of the ACO and SA was successfully used as a machine learning technique to generate classification rules; see [40,41]. Another promising area for using the hybrid metaheuristics is image processing. The hybrid metaheuristic algorithm combining the ACO, SA, and genetic algorithm was used for efficient contrast enhancement of images [42,43]. The combination of ACO and SA were used in a few cases also for the TSP. The authors of [44] enhanced their ACO algorithm with the idea inspired by the SA approach: the temperature is introduced as a new parameter decreasing in iterations. Then, solutions generated by individual ants in an iteration are selected to update the pheromone trails using the Metropolis criterion (the higher the temperature, the bigger probability for a solution to be selected, even if it is inferior). This approach increases the convergence speed. A different approach was adopted in [45]. The SA, along with the mutation operator, was used to increase the ant population diversity.

The authors of this article are not aware of any research which hybridizes ant colony optimization with simulated annealing for the dynamic traveling salesman problem. Although there are some publications combining the ACO and SA for the TSP, the ideas behind the integration of the both principles in these publications are different than the idea presented in this article.

## 3. Hybrid ACO Algorithm

In this section, a hybrid metaheuristic algorithm for the DTSP is proposed. The algorithm hybridizes two stochastic approaches: ant colony optimization (ACO) and simulated annealing (SA). The acronym ACO-SA is used for further reference. Both principles are first examined independently in Section 3.1 and Section 3.2, and then their hybridization is proposed in Section 3.3.

### 3.1. Ant Colony Optimization

The ACO, part of the ACO-SA algorithm, is based on the algorithm proposed by the authors for the multi-depot vehicle routing problem (MDVRP). This section adapts this algorithm to the DTSP; the MDVRP version can be found in [46]. The application of this algorithm to the DTSP instead of the MDVRP allows some simplifications which follow from using only a single colony of ants instead of multiple colonies, or missing constraints concerning the maximum length and/or capacity of vehicles. 

The key parameters of the ACO algorithm are in Table 1 along with a brief discussion. More information about their function and place in the algorithm can be found in the text below. Table 2 records all variables and symbols used in this section.

Algorithm 1 presents the ACO algorithm in pseudocode. For each iteration, including iteration zero (i=0, 1, 2,…,I), the algorithm repeats the same process in a loop (lines 3 to 34); the result of each loop is cost $C^{i}$ (line 33) and route $R^{i}$. (line 34) representing the i-th TSP solution. The first key step in each loop is the initialization of the pheromone matrix F (line 6). The details of this process are mentioned later in this section (see Algorithm 2). Note that in the zero iteration (i=0), route $R^{i-1}=R^{-1}$, which is undefined, is used as an input of the initialization function. It does not affect the functionality, as Algorithm 2 does not work with this parameter when i=0.

The process of finding a solution for the i-th TSP proceeds in a number of generations (lines 7 to 32). In each generation, heuristic information obtained in previous generations in the form of the pheromone matrix is used to generate a set of solutions; each solution (route $r^{a}$) is found for each ant in the colony ($a=1,2,\ldots ,N_{a}$) independently of one another (lines 9 to 25).

A solution for each ant is created based on the gradual selection of vertices from set $V^{i}$ and their insertion into route $r^{a}$ in a loop (lines 14 to 20). For this, set U is established, registering all vertices still missing in route $r^{a}$ (line 13). The loop ends when this set is empty, i.e., there are no more vertices in set U, which means that route $r^{a}$ contains all vertices from set $V^{i}$. Each vertex $U_{j}\in U$ is a candidate for insertion into route $r^{a}$; the probability of this insertion $p_{j}\left(U_{j}\right)$ (lines 15 to 16) is calculated according to Formula (4). The next vertex in the route is selected based on these probabilities (line 17) using a simple roulette wheel principle (see Algorithm 3 later in this section):

(4) $$p_{j}\left(U_{j}\right)=\frac{D_{kj}^{-\alpha }\cdot F_{kj}^{\beta }}{\sum_{U_{i}\in U}D_{ki}^{-\alpha }\cdot F_{ki}^{\beta }}\;\;\;\;\;\mathrm{for}\;\mathrm{all}\;U_{j}\in U$$

where $D_{kj}$ is the distance between the last vertex inserted into route $r^{a}$ (which is vertex $r_{k}^{a}$) and candidate vertex $U_{j}$ ($D_{kj}=\cos t\left(r_{k}^{a},\;U_{j}\right)$), $F_{kj}$ is a pheromone trail between the last vertex inserted into route and vertex $U_{j}$, $D_{ki}$ is the distance between the last vertex inserted into route and vertex $U_{i}\in U$ ($D_{ki}=\cos t\left(r_{k}^{a},\;U_{i}\right)$),$\;F_{ki}$ is a pheromone trail between the last vertex inserted into route and $U_{i}\in U$, α is the coefficient controlling the influence of the distance between vertices on the probability, and β is the coefficient controlling the influence of the pheromones on the probability. As can be seen, the distance is inversed, i.e., the bigger the distance, the lower the probability.

The best route $r^{a}$ of all ants ($a=1,2,\ldots ,N_{a}$.), i.e., the one with the lowest cost $c^{best}=\min\left(c^{1},c^{2},\ldots ,c^{N_{a}}\right)$, is stored as route $r^{best}$ (lines 23 to 25). If the best solution $r^{best}$ found in a generation is better than the best solution found so far in previous generations, this solution is saved as route $R^{i}$ with cost $C^{i}$ (lines 26 to 28).

Then, the pheromone matrix modification process starts; this process is composed of two phases. In the first phase (lines 29 to 30), the trails in the pheromone matrix evaporate; the speed is controlled by the pheromone evaporation coefficient ρ. In the second phase (lines 31 to 32), the pheromone matrix is updated according to best route $r^{best}$. Trails between neighboring vertices in route $r^{best}$ are intensified. The strength depends on the pheromone updating coefficient δ as well as on the quality of solution $c^{best}$ compared to the best-known solution $C^{i}$.

Although Algorithm 1, for the sake of simplicity, uses a constant number of generations to find a TSP solution (loop on lines 7 to 32), the real implementation enables two other termination conditions to end an individual iteration. The first one is the maximum time constraint; the second is the maximum specified number of generations without improving a solution (i.e., the number of generations in which the condition on line 26 is not met).

**Algorithm 1.** ACO algorithm for the DTSPAlgorithm_DTSP_ACO (*V*,*N_g_*,*N_a_*,*ρ*,*δ*,*τ*,*α*,*β*)
C=0R=∅**for** i=0 **to** *I* **do**      // Iterations   $C^{i}=\infty$   $R^{i}=\emptyset$   $F=\mathrm{Initialize}\text{\_}\mathrm{Pheromone}\text{\_}\mathrm{Matrix}\;(\left|V^{i}\right|,R^{i-1},\tau )$   **for** g=1 **to** *N_g_* **do**      // Generations    $c^{best}=\infty$    **for** a=1 **to** *N_a_* **do**    // Route for each ant     k=1     $c^{a}=0$     $r^{a}=\left\{V_{1}^{i}\right\}$     $U=V^{i}-\left\{V_{1}^{i}\right\}$     **while** U≠∅ **do**      **for each** $U_{j}\in U$ **do**       **compute** $p_{j}\left(U_{j}\right)=f\left(r_{k}^{a},V^{i},F,\alpha ,\beta \right)$      
$r_{k+1}^{a}=\mathrm{Select}\_\mathrm{Next}\_\mathrm{Vertex}\left(U,p_{1},p_{2},\ldots ,p_{\left|U\right|}\right)$      
$U=U-\left\{r_{k+1}^{a}\right\}$      
$c^{a}=c^{a}+\cos t\left(r_{k}^{a},\;r_{k+1}^{a}\right)$      
k=k+1     $r_{k+1}^{a}=V_{1}^{i}$     $c^{a}=c^{a}+\cos t\left(r_{k}^{a},\;r_{k+1}^{a}\right)$     **if** $c^{a}<c^{best}$ **then do**      
$c^{best}=c^{a}$      $r^{best}=r^{a}$     **if** $c^{best}<C^{i}$ **then do**      
$C^{i}=c^{best}$      
$R^{i}=r^{best}$    **for each** $F_{m,n}\in F$ **do**    // Evaporate pheromones     
$F_{m,n}=F_{m,n}\cdot \left(1-\rho \right)$    **for** k=1 to *N* **do**    // Update pheromones      $F_{r_{k}^{best},r_{k+1}^{best}}=F_{r_{k}^{best},r_{k+1}^{best}}+\delta \cdot \left(C^{i}/c^{best}\right)$   $C=C+C^{i}$   $R=R+\left\{R^{i}\right\}$**return***C,R*

Algorithm 2 presents the principle of the pheromone matrix initialization at the beginning of each i-th TSP iteration (see line 6 in Algorithm 1). In the first phase, all elements in N×N pheromone matrix F are set to 1 (lines 2 to 3); this value represents the initial pheromone strength of a connection between two vertices. In the second phase, a solution from the previous iteration (i−1) is used to update the matrix. This is based on an assumption following from the DTSP problem that only a small percentage of vertices changed from one iteration to another. Thus, a great part of the information from the solution found in the previous iteration is also valid in the current iteration. This information is integrated into the pheromone matrix by intensifying the trails between neighboring vertices in route $R^{i-1}$ (lines 5 to 6). The strength of this intensification is controlled by the pheromone initialization coefficient τ≥1. In the zero iteration (i=0) where there is no known route from the previous iteration, the second phase is skipped (see the condition on line 4). The second phase could also be skipped in case it is required to solve the DTSP problem as a number of independent TSP problems.

**Algorithm 2.** Pheromone matrix initialization
Initialize_Pheromone_Matrix (*N*,*R*^*i*−1^,*τ*)
  $F=\left(F_{m,n}\right)\in {\mathbb{R}}^{N\times N}$
   **for** $F_{m,n}\in F$ **do**
   
$F_{m,n}=1$   **if** i>0 **then do**    **for** k=1 to *N* **do**     
$F_{R_{k}^{i-1},R_{k+1}^{i-1}}=\tau$  **return** *F*

Algorithm 3 shows the principle of selecting the next vertex from set U based on probabilities $p_{1},p_{2},\ldots p_{\left|U\right|}$ (see line 17 in Algorithm 1). The algorithm uses the simple roulette wheel selection principle. RandU(a,b) is a pseudo-random number generator with uniform distribution ranging from a to b.

**Algorithm 3.** Selection of the next vertex in the route Select_Next_Vertex (*U*,*p_1_*,*p_2_*, ...*p_|U|_*)
  $p_{sum}=\underset{k}{\sum }p_{k}$
  
$p_{rnd}=\mathrm{RandU}\left(0,p_{sum}\right)$
   **for** k=1 **to** |*U*| **do**    **if** $p_{rnd}\leq \overset{k}{\underset{l=1}{\sum }}p_{l}$ **then do**     return *U_k_*

### 3.2. Simulated Annealing

The simulated annealing (SA) part of the ACO-SA algorithm is inspired by annealing in metallurgy, where this process is used to reduce the defects of material by way of heating and controlled cooling. The key idea behind the SA is to accept the worse solutions with some probability, thus expanding the search space explored for the global optimum. The SA can be used both for continuous and discrete state space.

The SA implementation presented in this section is only used for a single TSP problem instead of a series of TSP iterations. The reason for this is that it is needed for the hybridization with the ACO. To use it for the DTSP, the algorithm could be executed repeatedly with the solution from the previous iteration as an input.

Table 3 shows the key parameters of the SA algorithm; their function and place in the algorithm is mentioned below in more detail. Table 4 records all new symbols and variables used in this section; symbols in common with Section 3.1 can be found in Table 2.

Algorithm 4 shows the SA algorithm for the TSP in pseudocode. As input, route $R^{SA}$ enters the algorithm representing the first (initial) solution. This can be any feasible solution either found by another algorithm (e.g., by the nearest neighbor algorithm) or randomly generated (i.e., containing all vertices in random order). The algorithm works in generations (lines 4 to 19); during each generation, the same value is used for the temperature (starting with value $T_{max}$ in the first generation). When a generation ends, the temperature is lowered (line 18) and the next generation starts. When the temperature is lower than the minimum threshold $T_{min}$, the algorithm ends, returning the best solution found.

In each generation, a number of transformations and replacements are conducted (lines 6 to 17). The transformation of a current solution into a new solution (line 7) is a key part of the algorithm. This process is discussed later in this section (see Algorithm 5). The newly created solution replaces the original (lines 10 to 13) with a probability (line 9) calculated according to the Metropolis criterion (5). If the new solution is better than the original, it is always replaced. Otherwise, the probability of replacements depends on the difference in quality of both solutions and the current temperature. Higher temperatures increase the chances of accepting worse solutions; this happens more often in the initial phases (generations) of the algorithm.

(5) $$p\left(r^{SA}\to r^{SA^{’}}\right)=\begin{array}{cc} \{\;\begin{array}{l} 1\\ e^{-\frac{c^{SA^{’}}-c^{SA}}{T}} \end{array} & \begin{array}{l} \mathrm{for}\;c^{SA^{’}}\leq c^{SA}\\ \mathrm{otherwise} \end{array} \end{array}$$

The numbers of conducted transformations $n_{1}$ and replacements $n_{2}$ within a generation are limited by the parameters $n_{1max}$ and $n_{2max}$. Each generation ends when either $n_{1}$ or $n_{2}$ exceeds its permitted value. The best solution found during the execution of the algorithm is saved (lines 14 to 16) and returned when the algorithm ends (line 19).

**Algorithm 4.** SA algorithm for the TSP
Algorithm_TSP_SA (*V^i^*,*R^SA^*,*T_max_*,*T_min_*,*γ*,*n_1max_*,*n_2max_*)  $r^{SA}=R^{SA}$  $c^{SA}=C^{SA}=\overset{N}{\underset{k=1}{\sum }}\left|R_{k}^{SA}-R_{k+1}^{SA}\right|$  
$T=T_{max}$  **while** $T\geq T_{min}$ **do**        // Generations   $n_{1}=n_{2}=1$
   **while** $n_{1}\leq n_{1max}$ **and** $n_{2}\leq n_{2max}$ **do**    // Transformations
    $r^{SA^{’}}=\;\mathrm{Transform}\text{\_}\mathrm{Solution}\;(r^{SA},\left|V^{i}\right|,T,T_{max},T_{min})$
    
$c^{SA^{’}}=\overset{N}{\underset{k=1}{\sum }}\left|r_{k}^{SA^{’}}-r_{k+1}^{SA^{’}}\right|$    
$p\left(r^{SA}\to r^{SA^{’}}\right)=f\left(c^{SA},c^{SA^{’}},T\right)$    **if** $\mathrm{RandU}\left(0,1\right)\leq p\left(r^{SA}\to r^{SA^{’}}\right)$ **then do**     // Replacements      
$r^{SA}=r^{SA^{’}}$     
$c^{SA}=c^{SA^{’}}$     
$n_{2}=n_{2}+1$     **if** $c^{SA}<C^{SA}$ **then do**     
$R^{SA}=r^{SA}$     
$C^{SA}=c^{SA}$    
$n_{1}=n_{1}+1$   
T=γ⋅T **return** *C^SA^*,*R^SA^*


Table 5. Firstly, a random vertex from the original route is selected (except the first and the last vertices) to change its position (line 1); RandI(a,b) is a pseudo-random integer generator with uniform distribution ranging from a to b. Then, the range specifying the number of positions by which the selected vertex moves within the route is calculated (lines 2 to 3) using the RandN(μ,σ) function which is a pseudo-random number generator with normal distribution with a mean of μ=0 and a standard deviation of σ calculated according to Formula (6). This ensures that when the current temperature is not far from its maximum value, the selected vertex can be moved across the whole route ($\sigma =\frac{N}{3}$ for $T=T_{max}$), whereas when the current temperature is close to its minimum value, the selected vertex is moved only in the close vicinity around its position in the route (σ=1 for $T=T_{min}$). This principle ensures the extensive exploration of state space in the beginning phases of the algorithm and the tuning of the solution in the final phases. Finally, the vertex is moved in the transformed route $r^{SA^{’}}$ (lines 4 to 12) by a specified number of positions to the left (range<0) or the right (range>0).

(6) $$\sigma =\frac{\left(T-T_{min}\right)\cdot \left(\frac{N}{3}-1\right)}{T_{max}-T_{min}}+1$$

**Algorithm 5.** Solution transformationTransform_Solution (*r^SA^*,*N*,*T*,*T_max_*,*T_min_*)
  k=RandI(2,N)      // Vertex selection  $\sigma =f\left(N,T,T_{max},T_{min}\right)$  range=Round(RandN(0,σ))   // Range
  $r^{SA^{’}}=r^{SA}$  $i_{1}=i_{2}=k$   **for** j=1 **to** |*range*| **do    // Movement**   $i_{2}=i_{2}+\mathrm{Sgn}\left(range\right)$    **if** $i_{2}>N$ **then** $i_{2}=2$    **if** $i_{2}<2$ **then** $i_{2}=N$   $r_{i_{1}}^{SA^{’}}=r_{i_{2}}^{SA}$   $\;i_{1}=i_{2}$ $r_{i_{2}}^{SA^{’}}=r_{k}^{SA}$ **return** *r^SA’^*

### 3.3. Hybridization

In this section, hybridization of the ACO and SA algorithms is proposed. The basic idea behind this is as follows: the ACO algorithm (Section 3.1) is the key approach complemented by the SA algorithm (Section 3.2) which is used as a local optimization process. This process is applied to the best solution ($r^{best}$) found by the ants in a generation; this solution inputs the SA algorithm where it is possibly improved.

Table 5 shows the new parameters controlling this local optimization process. These parameters determine the generations in which the SA algorithm is executed. The first one ($sa_{freq}$) controls the frequency of executions, the second ($sa_{num}$) sets the last generation where the SA algorithm is executed (i.e., it is not executed in generations after the one given by this parameter).

Algorithm 6 shows the hybridization in pseudocode. The ACO part is simplified compared to the original, as shown in Algorithm 1. Instead, new procedures are used as follows:

- **Find_Route** (used on line 7 in Algorithm 6). This procedure covers the process of finding a route for each ant (it replaces lines 10 to 22 in Algorithm 1).
- **Get_Best_Route** (used on line 8 in Algorithm 6). This procedure selects the best solution found by ants (it replaces lines 23 to 25 in Algorithm 1).
- **Get_Better_Route** (used on line 11 in Algorithm 6). This procedure returns the better of two solutions in order to save the best solution found so far (it replaces lines 26 to 28 in Algorithm 1).
- **Evaporate_Pheromone_Matrix** (used on line 12 in Algorithm 6). This procedure evaporates the pheromone matrix as shown on lines 29 to 30 in Algorithm 1.
- **Update_Pheromone_Matrix** (used on line 13 in Algorithm 6). This procedure updates the pheromone matrix as shown on lines 31 to 32 in Algorithm 1.

The local optimization process in the form of the SA algorithm is located on lines 9 to 10 in Algorithm 6. This process is executed provided that the condition on line 9 is satisfied; this condition uses parameters as described in Table 5. If the condition is satisfied, the SA algorithm is executed; the best route found by ants in a generation ($r^{best}$) inputs the algorithm as an initial solution ($R^{SA}$). If the SA algorithm improves the initial solution, the solution $r^{best}$ is replaced by this new improved solution (if not, the solution returned by the SA algorithm is the same as the initial solution). The improved solution is used to update the pheromone matrix (line 13). Then, a new generation starts and the whole principle is repeated until the termination condition is met.

**Algorithm 6.** Hybrid ACO-SA algorithmAlgorithm_DTSP_ACO-SA (*V*,*N_g_*,*N_a_*,*ρ*,*δ*,*τ*,*α*,*β*,*T_max_*,*T_min_*,*γ*,*n_imax_*,*n_2max_*,*sa_freq_*,*sa_num_*)
  
R=∅   **for** i=0 **to** *I* **do**  // Iterations   $R^{i}=\emptyset$
   $F=\mathrm{Initialize}\text{\_}\mathrm{Pheromone}\text{\_}\mathrm{Matrix}(\left|V^{i}\right|,R^{i-1},\tau )$
    **for** g=1 **to** *N_g_* **do**   // Generations
     **for** a=1 **to** *N_a_* **do**     $r^{a}=\mathrm{Find}\_\mathrm{Route}(F,\alpha ,\beta )$    $r^{best}=\;\mathrm{Get}\text{\_}\mathrm{Best}\text{\_}\mathrm{Route}\;(r^{1},r^{2},\ldots ,r^{N_{a}})$     **if** $\left(g\;\mathrm{modulo}\;sa_{freq}\right)=0$ **and** $g\leq sa_{num}$ **then do**     $r^{best}=\;\mathrm{Algorithm}\text{\_}\mathrm{TSP}\text{\_}\mathrm{SA}(V^{i},r^{best},T_{max},T_{min},\gamma ,n_{1max},n_{2max})$     $R^{i}=\;\mathrm{Get}\text{\_}\mathrm{Better}\text{\_}\mathrm{Route}\;(R^{i}\;,r^{best})$    Evaporate_Pheromone_Matrix (*F*,*ρ*)    Update_Pheromone_Matrix (*F*,*δ*,*r^best^*)   $R=R+\left\{R^{i}\right\}$ **return** *R*

### 3.4. Computational Complexity

The computational complexity of the ACO algorithm is defined in Formula (7). It depends on the number of generations $N_{g}$ (linear dependence), the size of the population of ants $N_{a}$ (linear dependence), and the number of vertices N in the graph (quadratic dependence). The dependence on the number of vertices emerges three times on the left side of the formula: the left term $N^{2}$ is caused by finding a route for each ant (quadratic dependence), the middle term $N^{2}$ represents the pheromone evaporation process (quadratic dependence), and the right term N represents the pheromone updating process (linear dependence). However, both the pheromone evaporation and pheromone updating processes can be ignored (see the right side of the formula) as they are outside the loop for finding routes for ants. The quadratic complexity of finding a route for each ant is caused by consecutive insertion of N vertices into the route; the selection of each vertex is also linearly dependent on N as every vertex still missing in the route has to be considered (i.e., the probabilities of inserting all missing vertices have to be calculated).

(7) $$O\left(N_{g}\cdot \left(N_{a}\cdot N^{2}+N^{2}+N\right)\right)=O\left(N_{g}\cdot N_{a}\cdot N^{2}\right).\;$$

The computational complexity of the SA algorithm is defined in Formula (8). It depends on the number of generations $N_{sa}$ (linear dependence), the maximum number of transformations in a generation $n_{1max}$ (linear dependence), and the number of vertices N in the graph (linear dependence). The number of generations for the SA algorithm was not defined in the text above; it depends on the maximum and minimum temperature and cooling coefficient as shown in Formula (9). The maximum number of transformations $n_{1max}$ is used in Formula (8) instead of the maximum number of replacements $n_{2max}$ because the number of transformations is always equal to or greater than the number of replacements. The linear dependence on the number of vertices N represents the process of the solution transformation and its following evaluation.

(8) $$O\left(N_{sa}\cdot n_{1max}\cdot N\right)$$

(9) $$N_{sa}=⎡\frac{\log T_{min}-\log T_{max}}{\log\gamma }⎤\;$$

The final computational complexity of the proposed ACO-SA algorithm is defined in Formula (10) which combines Formulas (7) and (8). In each generation of the ACO part of the algorithm, routes for each ant need to be found (term $N_{a}\cdot N^{2}$), and then the SA algorithm is executed (term $N_{sa}\cdot n_{1max}\cdot N$).

(10) $$O\left(N_{g}\cdot \left(N_{a}\cdot N^{2}+N_{sa}\cdot n_{1max}\cdot N\right)\right)$$

## 4. Experiments and Results

This section presents experimental results of the ACO-SA algorithm. In the first part, the benchmark instances used in experiments are introduced. Then, the experiments and results are presented and evaluated and subsequently compared with other rival state-of-the-art metaheuristic algorithms. In the last part, various parameters and characteristics of the proposed algorithm are analyzed and discussed. The ACO-SA algorithm was implemented in C++ programming language using MS Visual Studio integrated development environment.

### 4.1. Benchmarks

The DTSP benchmark instances are based on the well-known TSPLIB symmetric problems [47]. These TSP problems represent the zero iteration $I_{0}$. Every successive iteration ($I_{1}$, $I_{2}$, …) is generated from the previous one by modifying positions of selected vertices; about 3% of vertices randomly change their positions in the next iteration. The same DSTP benchmark instances (created by the authors of [34]) were used for all algorithms to ensure even-handed comparison. The benchmark instances as well as the results are available for download at https://www.unob.cz/fvl/struktura/k111/Documents/DTSP_ACO-SA.zip.

Table 6 records the benchmark instances used. In total, there are six instances with various ranges of complexity: two small problems (N≤100), two medium problems (100<N≤400), and two large problems (N>400). In all cases, there are 11 iterations $I_{0}$ to $I_{10},$. i.e., the original TSP graph $I_{0}$ and 10 modified graphs $I_{1}$, $I_{2}$, …, $I_{10}$. The last column of Table 6 shows the representation of positions of vertices in a graph: it is either 2D Euclidean (the position of each vertex is represented by coordinates in two-dimensional space) or geographical (the position of each vertex is represented by longitude and latitude on Earth). This influences the way of computing the edge weights (distances between vertices) in the graph.

### 4.2. Experiments

Four different sets of experiments with all the benchmark instances were conducted as shown in Table 7; the sets are labeled with the letters *A*, *B*, *C*, and *D*. These sets of experiments vary one from another by (a) the algorithm used for the solution and (b) pheromone matrix initialization. In order to emphasize the improvement in results achieved due to hybridization, both the original ACO algorithm (sets *A* and *B*) and the hybridized ACO-SA algorithm (sets *C* and *D*) are used for the solution. The pheromone matrix initialization offers two possibilities: TSP and DTSP. The former (sets *A* and *C*) does not initialize the pheromone matrix (see line 6 in Algorithm 1) according to the solutions found in the previous iterations; this means that the DTSP problem is solved as a set of independent TSP problems. The latter (sets *B* and *D*) does initialize the pheromone matrix according to the solutions found in the previous iterations as shown in Algorithm 2. Thus, the difference when solving a dynamic TSP and a number of independent TSP problems can be emphasized.

Table 8 presents the settings of parameters of the algorithm used in experiments (for nomenclature, see Table 1, Table 3 and Table 5). As can be seen, some ACO parameters vary significantly for individual benchmark instances, especially ρ, τ, α, and β. One of the most important parameters is the pheromone evaporation coefficient ρ which differs even in the same instances for different sets of experiments. The SA parameters (for sets *C* and *D*) were set so that the execution time of the whole SA part takes about 40% of the entire execution time of the algorithm.

Table 9 and Table 10 show the results. Column T records the average execution time (in seconds) that the algorithm needs to solve a single TSP iteration. Every DTSP instance was solved 60 times; $C_{min\%}$ denotes the best solution found, $C_{avg\%}$ is the average value, and $\sigma_{\%}$ is the average standard deviation. The values $C_{min\%}$, $C_{avg\%}$, and $\sigma_{\%}$ are, for each instance, presented as percentages, as the distance to the optimal solution; optimal solutions for individual TSP iterations of instances were found using the famous Concorde TSP solver [13]. All the algorithms were executed on a computer configured as follows: CPU Intel i7-7700 3.5 GHz (4 cores), 32 GB RAM.

The results show the improvement of solutions when the initialization of the pheromone matrix according to the solutions from previous iterations is performed (compare the results from experiment *A* with *B* and *C* with *D*). It is clear that solving the DTSP problem is far better than solving its iterations as independent TSP problems. The improvement of solutions $C_{avg\%}$ over all benchmark instances is 6.13% (when comparing experiment *A* with *B*) and 3.84% (when comparing experiment *C* with *D*) respectively.

The enhancement of the original ACO algorithm by hybridization with the SA algorithm can be seen when comparing experiment *A* with *C* and *B* with *D*. The significant improvement is apparent—of course, at the cost of the longer execution time (about 40% longer, as mentioned above). The improvement calculated from the average solutions $C_{avg\%}$ over all benchmark instances when using the ACO-SA algorithm instead of the ACO algorithm is 10.57% (when comparing experiment *A* with *C*) and 8.29% (when comparing experiment *B* with *D*) respectively.

Logically, the best results were achieved in experiment *D* where the instances were solved as DTSP problems using the ACO-SA algorithm. The average solutions $C_{avg\%}$ for small and medium instances (*berlin52*, *kroA100*, *kroA200*, *gr202*) are not farther than 1% from the optimal solutions; for the large instances (*pcb442*, *gr666*), it is about 3%. The algorithm was able to find the minimum solutions $C_{min\%}$ as follows: equal to the optimal solution ($C_{min\%}=0\%$.)—for instance, *berlin52*, very close to the optimal solutions for instances *kroA100*, *kroA200*, and *gr202*, and about 2% distant from the optimal solutions for large instances *pcb442* and *gr666*. Moreover, in the case of instance *berlin52*, the optimal solution was reached in all 60 executions ($C_{min\%}=C_{avg\%}=0\%$).

### 4.3. Comparison

The results achieved in experiment *D* were compared with four other state-of-the-art metaheuristic algorithms, two of them based on the ACO principle, the other two on the PSO principle. These are as follows:

- **ACS**: ant colony system [48].
- **PACO**: population-based ant colony optimization [49].
- **DPSO-Ho**: discrete particle swarm optimization with homogeneous parameter values [35].
- **DPSO-He**: discrete particle swarm optimization with heterogeneous parameter values [34].

The same benchmark instances were used for all the algorithms to ensure fair comparison. Furthermore, the same size of population/swarm ($N_{a}$) as well as the number of generations ($N_{g}$) were used; this means that the same number of solutions were generated and evaluated during the PSO or ACO part of the algorithm (not counting transformations of the solution via simulated annealing). Moreover, various numbers of generations were used for individual instances to compare the quality of the solutions provided by the algorithms depending on the number of generations used.

Table 11 presents the settings of the pheromone evaporation coefficient of the ACO-SA algorithm for individual instances and different numbers of generations; this is the only parameter which changes when solving an instance using a different number of generations, as it controls the convergence speed. The remaining parameters are the same as those used in experiment *D* (see Table 8).

The evaluation and comparison of results provided by the ACO-SA and the rival algorithms is in Table 12. For each instance and number of generations (i.e., an experiment), the table compares the average solutions $C_{avg\%}$ and the average standard deviation $\sigma_{\%}$ (the latter only in the cases of the DPSO and ACO-SA algorithms). In each experiment, the ACO-SA algorithm was executed 60 times, providing 60 independent solutions that were averaged. The results for the rival algorithms were taken from [34]. The best solution for each row is marked in bold. The last row of Table 12 shows the average value of each column.

The results obtained by the ACO-SA algorithm outperform other approaches. With the single exception of instance *pcb442* and $N_{g}=4352$, ACO-SA provided better (or the same) average solutions in all cases. The average value of $C_{avg\%}$ calculated from all experiments is about 1.45% better compared to the second-best algorithm (PACO). Additionally, the standard deviation of the solutions is lower in most cases compared to both DPSO approaches; this shows greater stability of solutions in experiments (which are, at the same time, better).

In particular, the ACO-SA algorithm is especially strong, i.e., providing high-quality solutions, when using the low number of generations. For example, in the case of instance *gr666* and $N_{g}=384$, the solutions are 4.46% away from the optimal solution, whereas it is 5.89% in case of the PACO algorithm and more than 9% in case of other algorithms (note, for instance, that the results achieved on instance *gr666* with $N_{g}=768$ are better than the results achieved via all other algorithms for $N_{g}=6144$). On average, the ACO-SA algorithm provides about 1.79% better solutions than PACO when using the lowest number of generations in Table 12 for each instance ($N_{g}=384$ for *gr666*, $N_{g}=272$ for *pcb442*, etc.); however, in the case of DPSO-Ho, it is more than 10%.

Table 13 compares the time needed for optimization of the algorithms DPSO-Ho and DPSO-He with ACO-SA. The comparison is for illustration only, as different computer configurations were used in the experiments (optimization times were taken from [34] for DPSO algorithms). Despite this, it is apparent that the ACO-SA algorithm is comparable to the small and medium instances. However, it takes longer to optimize it when using the same population size as well as the same number of generations for the large instances—in most cases, but not all of them. The optimization is faster, for example, in the case of instance *gr666* and $N_{g}=384$, and is comparable for $N_{g}=768$. 

**Figure 1:**
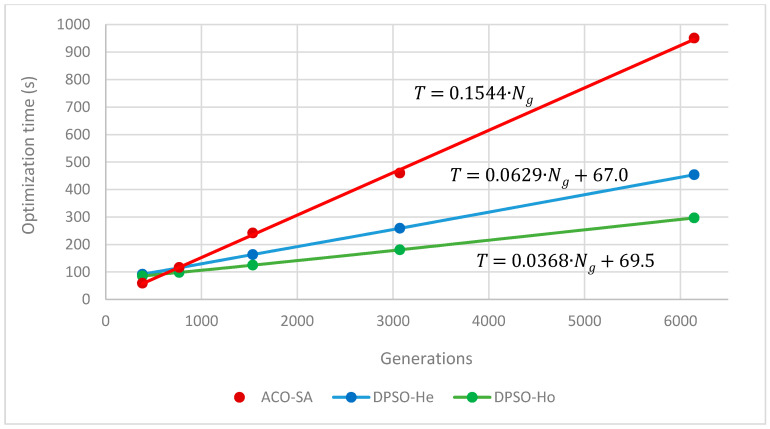
Dependence of optimization time on the number of generations (instance *gr666*).

The linear dependence of the algorithms on the number of generations can be seen in Figure 1 using the example of instance *gr666*. For the ACO-SA algorithm, the optimization time can easily be estimated using formula: $T=T_{1}\cdot N_{g}$ where $T_{1}$ is the time needed for a single generation. This is not true for the DPSO algorithms where the extra time for preparation before the first generation starts is required: $T=T_{1}\cdot N_{g}+T_{0}$. Values of $T_{1}$ and $T_{0}$ for the algorithms for instance *gr666* are shown in Figure 1.

### 4.4. Analysis and Discussion

In this section, various features and parameters of the ACO-SA algorithm are examined (convergence speed, population diversity, improvement by simulated annealing, dependence of optimization time on the number of vertices, and further possibilities to improve the solution).

#### 4.4.1. Convergence

**Figure 2:**
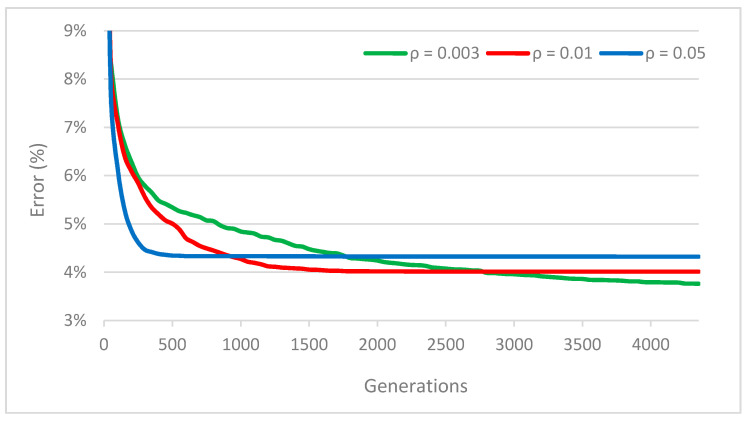
Solution convergence in dependence on the pheromone evaporation coefficient (instance *pcb442*).

First, the influence of the pheromone evaporation coefficient ρ is discussed. This coefficient controls the speed of convergence to a local optimum. Figure 2 illustrates this on the zero TSP iteration $I_{0}$ of instance *pcb442* for values of ρ as follows: ρ=0.003, ρ=0.01, ρ=0.05. The graph shows the progress of the solution quality during optimization (averaged from 60 optimization trials). As is obvious, the bigger values of ρ cause faster convergence but lower final solution quality. The values of ρ are taken from experiment *D* and they were set so that the results would be as good as possible with the number of generations available ($N_{g}=4352$, $N_{g}=1088$, and $N_{g}=272$ respectively). Note, for example, that for ρ=0.05, the solution reaches its final quality somewhere close to the 300th generation.

#### 4.4.2. Population Diversity

In this section, the diversity of solutions in a population of ants, and its progress with increasing numbers of generations, is studied on the zero iteration $I_{0}$ of instance *gr202*. In general, large diversity is desired in the early phases of optimization because it prevents solutions from getting stuck at a not-so-good local optimum. On the other hand, it should be low towards the end of optimization to tune the solution to a good local optimum (or even global optimum).

The diversity of solutions in the population of a particular generation is expressed by Shannon entropy H according to Formula (11) where $p_{ij}$ is the probability that edge $E_{ij}$ between vertices $V_{i}$ and $V_{j}$ (i≠j) is part of any solution in the population ($E_{ij}=E_{ji}$). This probability can be calculated based on the numbers of occurrences of edges in the solutions (see Formula (12)). The occurence function returns the number of times edge $E_{ij}$ is part of solution routes in a population of size $N_{a}$. The denominator expresses the total number of edges of which the routes are comprised.

(11) $$H=-\overset{N}{\underset{i=2}{\sum }}\overset{i-1}{\underset{j=1}{\sum }}p_{ij}\cdot \log_{2}p_{ij}$$

(12) $$p_{ij}=\frac{occurence\left(E_{ij}\right)}{N_{a}\cdot N}$$

**Figure 3:**
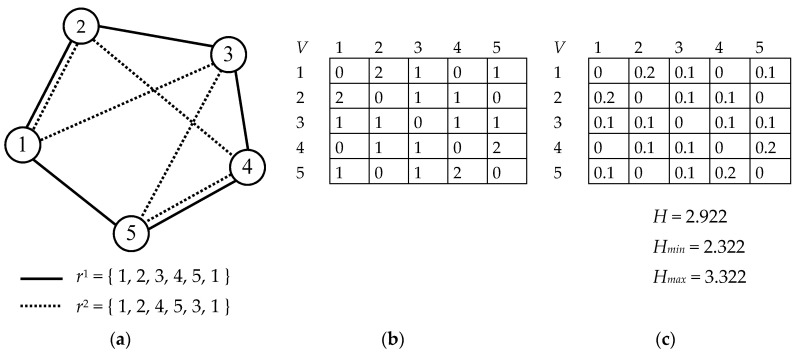
Principle of entropy calculation: (**a**) TSP example; (**b**) edge occurrences; (**c**) edge probabilities.

Figure 3a shows the principle of the entropy calculation on a simple TSP example with 5 vertices (N=5). The population is composed of two ants: $N_{a}=2$. As can be seen, there are two common edges for both solutions ($E_{12}$, $E_{45}$) and 6 distinct edges ($E_{13}$, $E_{15}$, $E_{23}$, $E_{24}$, $E_{34}$, $E_{35}$); thus, $occurence\left(E_{12}\right)=occurence\left(E_{45}\right)=2$ and $occurence\left(E_{13}\right)=\ldots =1$. The values can be also represented in a table as shown in Figure 3b; note that only half of the values in the table in Figure 3b (the values shaded in grey) are used to calculate entropy (because $E_{ij}=E_{ji}$). The probabilities $p_{ij}$ are calculated in the table in Figure 3c, and the entropy for this population is H=2.922.

It is also easy to calculate the minimum and maximum limits of entropy for each instance and population size (see Formulas (13) and (14)). The minimum entropy $H_{min}$ represents the population in which all solutions are exactly the same (for example, $r^{1}=r^{2}=\left\{1,\;2,\;3,\;4,\;5,\;1\right\}$ in case of the TSP in Figure 3; $H_{min}=2.322$). On the other hand, the maximum entropy $H_{max}$ represents the population in which no edge appears two or more times in every solution (for example, $r^{1}=\left\{1,\;2,\;3,\;4,\;5,\;1\right\}$ and $r^{2}=\left\{1,\;3,\;5,\;2,\;4,\;1\right\}$ in the case of the TSP in Figure 3; $H_{max}=3.322$).

(13) $$H_{min}=\log_{2}\frac{1}{N}$$

(14) $$H_{max}=\log_{2}\frac{1}{N_{a}\cdot N}$$

**Figure 4:**
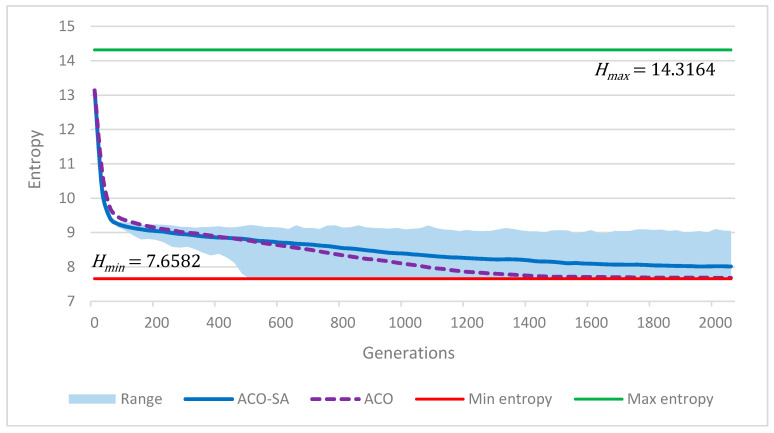
Population diversity (instance *gr202*).

The development of entropy during optimization in case of instance *gr202* is shown in Figure 4; the minimum and maximum limits of entropy are also emphasized. The blue curve is the averaged value computed from 60 optimization trials using the ACO-SA algorithm, the light blue area represents the range of entropy in these 60 trials. At the beginning, the entropy is close to its maximum limit, then it drops fast to the point where it decreases almost linearly towards its minimum limit. Indeed, a value of entropy close to the minimum limit is reached in some cases; and in some of them, this minimum entropy was reached at somewhere around the 500th generation, which is about one quarter of optimization. The dashed violet curve shows the same task but this time using the ACO algorithm for optimization (i.e., without simulated annealing). It is clear that the diversity of the population drops faster in this case. Thus, the simulated annealing also contributes to maintaining the diversity of the population.

In trials where the entropy drops very close to its minimum limit, the solution converges to a local (or global) optimum, and the probability that it would be further improved in the next generations is very low. Therefore, the entropy can be used as a good criterion to terminate the optimization before it reaches the last generation $N_{g}$. This idea was tested on instance *gr202*; the optimization was terminated when the entropy was less than 1% away from the minimum limit. The average optimization time in 60 trials was reduced by 68% (from 28.5 s to 9.1 s) while, at the same time, the average solution remained the same. This means that the average number of generations used in a trial was reduced from 2048 to 655. This big speedup is caused particularly in DTSP iterations ($I_{1}$ to $I_{10}$) in which the speed of convergence is faster than in iteration $I_{0}$ due to the pheromone matrix initialization.

#### 4.4.3. Simulated Annealing Improvements

In this section, the role of simulated annealing in the ACO-SA algorithm is examined. The SA algorithm is applied to the best solution in a generation which is then used to update the pheromone matrix (see points 31 and 32 of Algorithm 1). The best solution can be improved or, if not, it remains the same.

**Figure 5:**
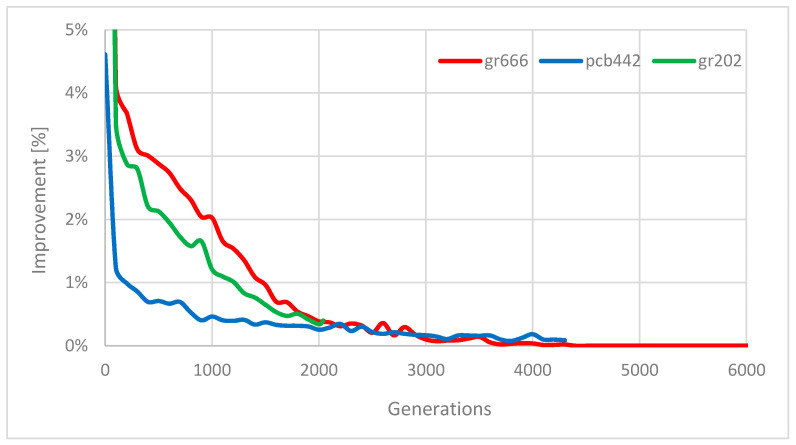
Solution improvements by simulated annealing.

The average improvements of the best solution in dependence on a current generation for instances *gr666* ($N_{g}=6144$), *pcb442* ($N_{g}=4352$), and *gr202* ($N_{g}=2048$) are shown in Figure 5. The improvement is high in the first phases of the optimization (it is higher than 20% in the first generations for instances *gr202* and *gr666*), the effect of which is the fast convergence. Then, the improvement decreases at different speeds for individual instances. In case of instances *gr202* and *pcb442*, the improvement is non-zero during the entire optimization. In the case of instance gr666, it is zero in the last third of the optimization (note the purpose of parameter $sa_{num}$; for this reason, in case of instance *gr666*, this parameter was set to $sa_{num}=\frac{2}{3}\cdot N_{g}$ in order to save the execution time). In general, the behavior of simulated annealing varies for each instance to be solved.

#### 4.4.4. Time Dependence

**Figure 6:**
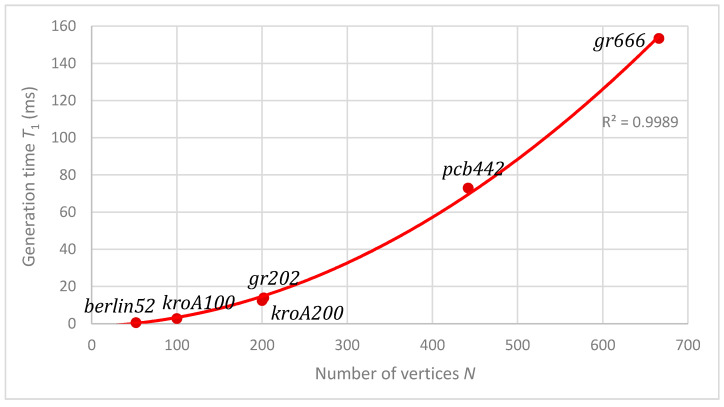
Dependence of optimization time on the number of vertices.

The linear dependence of the algorithm on the number of generations was shown in Section 4.3 (see Figure 1). In this section, the dependence on the number of vertices is presented. For all six benchmark instances, the average time $T_{1}$ needed for a single generation was calculated, and its dependence on the number of vertices N is shown in Figure 6. The quadratic dependence is obvious; this confirms Formula (10) in Section 3.

#### 4.4.5. Further Possibilities to Improve the Solution

Although the ACO-SA algorithm executed on the benchmark instances outperforms the other metaheuristic algorithms, there are more ways to improve the results. One of them is the application of exact optimization techniques to the results after the optimization. In this section, this idea is tested using the well-known *k*-opt optimization technique [50]. This technique was applied to all solutions (i.e., 60 solutions per instance and number of generations) provided by the ACO-SA algorithm (see Table 12).

Table 14 shows the results for k=2, 3, 4, 5. The improvement in the solutions is tangible. In the case of instance *gr202*, for example, the 2-opt optimization improved solution $C_{avg\%}$ from 2.215% to 1.523%. In general, the size of improvement corresponds to the size of the solution gap to the optimum. It seems that using k=2 or k=3 is reasonable enough; higher values (k>3) have only a small further effect. Table 15 shows the average execution time needed for *k*-opt post-processing of a single TSP iteration (in seconds). For the small and medium instances, the execution time is insignificant; for the large instances (*pcb442* and *gr666*), it is minor compared to the total optimization time (e.g., about 3% of the total time in case of instance *gr666*, $N_{g}=6144$ and k=2). However, this process could be made significantly faster with the help of a few simple improvements (e.g., limiting the search to a number of the nearest neighbors) [51].

## 5. Conclusions

The ACO-SA algorithm proposed in this article uniquely combines two metaheuristic principles: ant colony optimization and simulated annealing. Four different experiments on selected benchmark instances (*A*, *B*, *C*, *D*) were conducted. The significant improvement in solutions when using the hybridized ACO-SA algorithm instead of the original ACO is apparent (compare the results in Section 4.2 of experiment *A* or *B* with *C* or *D*). On the other hand, the comparison of results from experiment *A* or *C* with *B* or *D* clearly proves the importance of incorporating the information about the changes in dynamic environment into the algorithm in the form of the pheromone matrix initialization.

The results achieved by the proposed ACO-SA algorithm significantly outperform the results of the four other metaheuristic approaches based on ant colony optimization or discrete particle swarm optimization. From the time point of view, for the small and medium problems, the time needed for optimization is comparable with these methods; this is true even for large problems with low numbers of generations. However, for large problems with higher numbers of generations, the time needed for optimization is longer than in the case of rival methods. This is caused by the quadratic dependence of the ACO-SA algorithm on the problem size. Because of the time as well as memory reasons (memory requirements are also quadratic dependent on the number of vertices), the proposed ACO-SA algorithm is not suitable for very large problems where N>1000.

The behavior of the ACO-SA algorithm can be controlled by several key parameters (the one most significantly influencing the behavior is the pheromone evaporation coefficient—see Section 4.4.1). Unfortunately, the optimal settings of the values of these parameters cannot be determined analytically in most cases as they are problem dependent (this can be true even for problems of the same or similar size and, to some extent, even for different iterations of the same DTSP). This means that finding the best values of parameters for a given problem sometimes requires a massive number of trials, which is, of course, time-consuming (although the empirical knowledge can shorten this time significantly). For this reason, it is still possible (and even probable) that the values of the parameters used to provide results in Section 4.2 (see Table 8) are still not optimally selected; thus, different values may be found so that the algorithm would provide even better results. 

Future research of the authors, besides that dealing with other issues, will be devoted to resolving the drawback mentioned in the previous paragraph. In particular, the following issues will be pursued:

- Examination of the possibility of heterogeneous parameters. This means that the parameters would not have constant values during the whole optimization, but their values may change if necessary, based on the current situation. As an example, the pheromone evaporation coefficient will serve: at the beginning of the optimization, the higher values would ensure faster convergence; then, its value would gradually decrease based on the current state of the optimization (which can be, for example, assessed based on the value of entropy in the population—see Section 4.4.2). Both the upper and bottom limits of the coefficient will be adjusted automatically so that the user does not have to search for them.
- Examination of the influence of the time devoted to the SA part of the algorithm on the quality of the results. In this article, the parameters of the SA were set for all benchmark instances in such a way that the simulated annealing would take about 40% of the whole optimization time (this was the condition determined by the authors before the experiments). It is expected that better solutions would be provided if a longer time were reserved for the SA (e.g., 50%, 60%, 80%)—but of course at the expense of a longer execution time. The improvement in solutions in dependence on the time distribution between the ACO and SA parts requires further study.
- Hybridization of the ACO-SA algorithm with the local search optimization (e.g., *k*-opt optimization technique). This means that the local search optimization is used not only at the end of the algorithm as shown in Section 4.4.5 but also during the optimization process in selected generations. The local search and the SA process might complement each other.
- Extension of the ACO-SA algorithm for other optimization problems related to the TSP. The algorithm can be relatively easily modified to solve NP-hard problems such as vehicle routing problems (VRP), the multi-depot vehicle routing problem (MDVRP), and their variants.

## Figures and Tables

**Table 1:** Key parameters of the ant colony optimization (ACO) algorithm.

$N_{g}$	**Number of generations of the algorithm.** In each traveling salesman problem (TSP) iteration, the same number of generations is executed; in each generation, solutions are generated based on the heuristic information gathered in previous generations.
$N_{a}$	**Number of ants in a colony**. For each ant in each generation, an independent solution is found. In total, $N_{g}\cdot N_{a}$ solutions are generated and evaluated in a single TSP iteration of the algorithm.
ρ	**Pheromone evaporation coefficient**. This coefficient controls the speed of the pheromone evaporation process, which is a key principle ensuring that solutions of lower quality from earlier generations lose their influence in the pheromone matrix in favor of higher quality solutions from recent generations. This coefficient has great impact on the speed of convergence to a local optimum; the correct setting is of significance for any dynamic traveling salesman problem (DTSP).
δ	**Pheromone updating coefficient**. This coefficient controls the influence of the best solution found in each generation on the pheromone matrix when updating the pheromone trails according to this solution.
τ	**Pheromone initialization coefficient**. This coefficient controls the initial strength of the pheromone trails in each DTSP iteration according to the best solution found in the previous iteration. The main idea behind this is the knowledge that only a small portion of vertices changes from one TSP iteration to another.
α	**Distance probability coefficient**. This coefficient controls the influence of the distance between vertices when calculating the probability to select the next vertex on the route.
β	**Pheromone probability coefficient**. This coefficient controls the influence of the strength of pheromone trails between vertices when calculating the probability to select the next vertex on the route.

**Table 2:** Variables and symbols used in Section 3.1.

I	Number of iterations of the DTSP.
N	Number of vertices in each TSP.
V	A set of sets of all vertices of the DTSP; $V=\left\{V^{0},V^{1},\ldots ,V^{I}\right\}$.
$V^{i}$	A set of vertices of the i-th TSP iteration; $V^{i}=\left\{V_{1}^{i},V_{2}^{i},\ldots ,V_{N}^{i}\right\}$, i=0, 1, 2, …, I.
$V_{k}^{i}$	k-th vertex of the i-th TSP iteration; i=0, 1, 2, …, I, k=1, 2, …, N.
C	Total cost of thDTSP solution.
$C^{i}$	Cost of the i-th TSP solution; i=0, 1, 2, …, I.
$c^{a}$	Cost of a solution found by the a-th ant in the colony; $a=1,\;2,\;\ldots ,\;N_{a}$.
$c^{best}$	The lowest cost of all solutions found by ants in a generation.
R	A set of all routes for the DTSP; $R=\left\{R^{0},R^{1},\ldots ,R^{I}\right\}$.
$R^{i}$	A route for the i-th TSP;$\;R^{i}=\left\{R_{1}^{i},R_{2}^{i},\ldots ,R_{N}^{i},R_{N+1}^{i}\right\}$, i=0, 1, 2, …, I.
$R_{k}^{i}$	k-th vertex in a route of the i-th TSP; i=0, 1, 2, …, I, k=1, 2, …, N+1.
$r^{a}$	A route found by the a-th ant in the colony;$\;r^{a}=\left\{R_{1}^{a},R_{2}^{a},\ldots ,R_{N}^{a},R_{N+1}^{a}\right\}$, $a=1,\;2,\;\ldots ,\;N_{a}$.
$r_{k}^{a}$	k-th vertex in a route of the a-th ant; $a=1,\;2,\;\ldots ,\;N_{a}$, k=1, 2, …, N+1.
$r^{best}$	The route with the lowest cost found by ants in a generation.
U	A set of alvertices still missing in a route.
$U_{j}$	A selected vtex still missing in a route; $U_{j}\in U$.
$p_{j}\left(U_{j}\right)$	Probability that vertex *U_j_* will be chosen as the next vertex in route *r^a^*
F	An N×N pheromone matrix.
$F_{m,n}$	An element of matrix F; m=1, 2, …, N, n=1, 2, …, N.

**Table 3:** Key parameters of the SA algorithm.

**$T_{max}$**	**Maximum temperature. The initial value of the temperature used in the first generation.**
$T_{min}$	**Minimum temperature**. The threshold value of the temperature. When the current temperature drops below this threshold, the algorithm ends.
γ	**Cooling coefficient**. This coefficient controls the speed of temperature cooling in successive generations (0<γ<1).
$n_{1max}$	**Maximum number of transformations in a generation**. In each generation, a current solution is repeatedly transformed into a new solution. These coefficients control the number of those transformations in each generation.
$n_{2max}$	**Maximum number of replacements in a generation**. In each generation, a newly created (transformed) solution can replace the original solution with a probability. These coefficients control the number of those replacements in each generation.

**Table 4:** Variables and symbols used in Section 3.2.

$C^{SA}$	Cost of the initial and final TSP solution.
$c^{SA}$	Cost of the current solution.
$c^{SA^{’}}$	Cost of the solution transformed from the current solution.
$R^{SA}$	A route of the initial and final TSP solution; $R^{SA}=\left\{R_{1}^{SA},R_{2}^{SA},\ldots ,R_{N}^{SA},R_{N+1}^{SA}\right\}$.
$R_{k}^{SA}$	k-th vertex in a route of solution $R^{SA}$; k=1, 2, …, N+1.
$r^{SA}$	A route of the current solution;$\;r^{SA}=\left\{r_{1}^{SA},r_{2}^{SA},\ldots ,r_{N}^{SA},r_{N+1}^{SA}\right\}$.
$r_{k}^{SA}$	k-th vertex in a route of the current solution; k=1, 2, …, N+1.
$r^{SA^{’}}$	A route of the transformed solution;$\;r^{SA^{’}}=\left\{r_{1}^{SA^{’}},r_{2}^{SA^{’}},\ldots ,r_{N}^{SA^{’}},r_{N+1}^{SA^{’}}\right\}$.
$r_{k}^{SA^{’}}$	k-th vertex in a route of the transformed solution; k=1, 2, …, N+1.
T	Current temperature.
$n_{1}$	Current number of transformations in a generation.
$n_{2}$	Current number of replacements in a generation.
$p\left(r^{SA}\to r^{SA^{’}}\right)$	Probability that the transformed solution $r^{SA^{’}}$ replaces the original solution $r^{SA}$.

**Table 5:** New parameters of the ACO-SA algorithm.

$sa_{freq}$	**Execution frequency of the SA algorithm**. This parameter determines the rate of executions of the SA algorithm in generations, i.e., the SA algorithm is executed in every $sa_{freq}$-th generation.
$sa_{num}$	**Number of generations where the SA algorithm is executed**. This parameter determines the generations ($g=1,2,\ldots ,sa_{num}$) where the SA algorithm is executed (not the number of executions).

**Table 6:** Benchmark instances.

Benchmark Instance	Number of Vertices (N)	Number of Iterations (I)	Position Representation
*berlin52*	52	11	2D Euclidean
*kroA100*	100	11	2D Euclidean
*kroA200*	200	11	2D Euclidean
*gr202*	202	11	Geographical
*pcb442*	442	11	2D Euclidean
*gr666*	666	11	Geographical

**Table 7:** Experiment sets.

Experiment Set	Algorithm Used	Pheromone Matrix Initialization
*A*	ACO	TSP
*B*	ACO	DTSP
*C*	ACO-SA	TSP
*D*	ACO-SA	DTSP

**Table 8:** Settings of parameters for experiments.

Instance	Set	ACO Parameters							SA Parameters					ACO-SA	
		$N_{g}$	$N_{a}$	ρ	δ	α	β	τ	$T_{max}$	$T_{min}$	γ	$n_{1max}$	$n_{2max}$	$sa_{freq}$	$sa_{num}$
*berlin52*	*A*	1664	32	0.006	1	1	1	0	–	–	–	–	–	–	–
	*B*	1664	32	0.004	1	1	1	10	–	–	–	–	–	–	–
	*C*	1664	32	0.001	1	1	3	0	1	0.1	0.8	50	5	1	1664
	*D*	1664	32	0.001	1	1	3	10	1	0.1	0.8	50	5	1	1664
*kroA100*	*A*	1600	64	0.006	1	1	2	0	–	–	–	–	–	–	–
	*B*	1600	64	0.005	1	1	2	20	–	–	–	–	–	–	–
	*C*	1600	64	0.006	1	1	2	0	1	0.02	0.9	60	5	1	1600
	*D*	1600	64	0.006	1	1	2	30	1	0.02	0.9	60	5	1	1600
*kroA200*	*A*	2560	80	0.007	1	1	4	0	–	–	–	–	–	–	–
	*B*	2560	80	0.007	1	1	4	50	–	–	–	–	–	–	–
	*C*	2560	80	0.008	1	1	4	0	1	0.03	0.9	200	10	1	2560
	*D*	2560	80	0.008	1	1	4	50	1	0.03	0.9	200	10	1	2560
*gr202*	*A*	2048	101	0.1	1	1	1	0	–	–	–	–	–	–	–
	*B*	2048	101	0.1	1	1	1	20	–	–	–	–	–	–	–
	*C*	2048	101	0.13	1	1	1	0	1	0.03	0.9	200	10	1	2048
	*D*	2048	101	0.13	1	1	1	500	1	0.03	0.9	200	10	1	2048
*pcb442*	*A*	4352	104	0.005	1	2	5	0	–	–	–	–	–	–	–
	*B*	4352	104	0.001	1	2	5	20	–	–	–	–	–	–	–
	*C*	4352	104	0.003	1	2	5	0	1	0.01	0.9	500	20	1	4352
	*D*	4352	104	0.003	1	2	5	20	1	0.01	0.9	500	20	1	4352
*gr666*	*A*	6144	112	0.02	1	1	2.5	0	–	–	–	–	–	–	–
	*B*	6144	112	0.007	1	1	2.5	500	–	–	–	–	–	–	–
	*C*	6144	112	0.11	1	1	2.5	0	10	0.1	0.9	1000	20	1	4096
	*D*	6144	112	0.11	1	1	2.5	500	10	0.1	0.9	1000	20	1	4096

**Table 9:** Results of experiments *A* and *B*.

Instance	Experiment *A*				Experiment *B*			
	T (s)	$C_{min\%}$	$C_{avg\%}$	$\sigma_{\%}$	T (s)	$C_{min\%}$	$C_{avg\%}$	$\sigma_{\%}$
*berlin52*	0.49	0.00%	0.15%	0.27%	0.48	0.01%	0.02%	0.03%
*kroA100*	2.47	0.60%	1.96%	0.75%	2.61	0.39%	0.68%	0.21%
*kroA200*	17.49	1.50%	3.03%	0.85%	18.73	0.77%	1.45%	0.33%
*gr202*	17.89	1.37%	3.14%	0.88%	18.02	1.22%	2.75%	0.74%
*pcb442*	179.45	4.46%	6.76%	0.79%	183.33	4.18%	5.05%	0.33%
*gr666*	545.88	4.89%	6.76%	0.95%	557.08	4.40%	5.71%	0.58%

**Table 10:** Results of experiments *C* and *D*.

Instance	Experiment *C*				Experiment *D*			
	T (s)	$C_{min\%}$	$C_{avg\%}$	$\sigma_{\%}$	T (s)	$C_{min\%}$	$C_{avg\%}$	$\sigma_{\%}$
*berlin52*	0.90	0.00%	0.08%	0.31%	0.90	0.00%	0.00%	0.00%
*kroA100*	4.34	0.06%	0.39%	0.19%	4.51	0.03%	0.17%	0.09%
*kroA200*	30.85	0.64%	1.37%	0.41%	32.02	0.21%	0.63%	0.23%
*gr202*	27.79	0.35%	1.36%	0.70%	28.53	0.30%	0.97%	0.44%
*pcb442*	317.45	2.43%	4.17%	0.66%	312.59	1.85%	2.56%	0.30%
*gr666*	948.34	2.06%	3.85%	0.89%	951.12	2.09%	3.06%	0.51%

**Table 11:** Settings of the pheromone evaporation coefficient.

Instance											
*berlin52*		*kroA100*		*kroA200*		*gr202*		*pcb442*		*gr666*	
$N_{g}$	ρ	$N_{g}$	ρ	$N_{g}$	ρ	$N_{g}$	ρ	$N_{g}$	ρ	$N_{g}$	ρ
104	0.07	100	0.01	160	0.033	128	0.29	272	0.05	384	0.16
416	0.016	400	0.007	640	0.03	512	0.2	1088	0.01	768	0.14
1664	0.001	1600	0.006	2560	0.008	2048	0.13	4352	0.003	1536	0.13
–	–	–	–	–	–	–	–	–	–	3072	0.11
–	–	–	–	–	–	–	–	–	–	6144	0.11

**Table 14:** Results after *k*-opt optimization.

Instance	$N_{g}$	ACO-SA	2-opt	3-opt	4-opt	5-opt
*berlin52*	104	0.057%	0.032%	0.030%	0.030%	0.030%
	416	0.006%	0.006%	0.004%	0.004%	0.004%
	1664	0.000%	0.000%	0.000%	0.000%	0.000%
*kroA100*	100	0.745%	0.470%	0.385%	0.375%	0.372%
	400	0.287%	0.227%	0.201%	0.199%	0.199%
	1600	0.168%	0.163%	0.154%	0.154%	0.154%
*kroA200*	160	1.186%	0.858%	0.828%	0.817%	0.816%
	640	0.811%	0.672%	0.656%	0.638%	0.638%
	2560	0.627%	0.507%	0.492%	0.488%	0.488%
*gr202*	128	2.215%	1.523%	1.465%	1.454%	1.447%
	512	1.335%	1.041%	1.009%	1.000%	0.999%
	2048	0.973%	0.790%	0.770%	0.766%	0.762%
*pcb442*	272	3.071%	2.249%	2.186	2.164%	2.156%
	1088	2.826%	2.069%	2.029%	2.016%	2.013%
	4352	2.564%	1.921%	1.869%	1.846%	1.842%
*gr666*	384	4.457%	3.335%	3.230%	3.190%	3.166%
	768	3.572%	2.831%	2.763%	2.738%	2.723%
	1536	3.487%	3.018%	2.970%	2.946%	2.923%
	3072	3.224%	2.780%	2.723%	2.705%	2.691%
	6144	3.058%	2.567%	2.526%	2.510%	2.501%
**Average values**		1.733%	1.353%	1.315%	1.302%	1.296%

**Table 15:** Execution time of *k*-opt optimization.

Instance	2-opt (s)	3-opt (s)	4-opt (s)	5-opt (s)
*berlin52*	0.004	0.005	0.005	0.006
*kroA100*	0.012	0.020	0.025	0.030
*kroA200*	0.127	0.181	0.228	0.274
*gr202*	0.189	0.255	0.303	0.352
*pcb442*	5.214	6.124	6.858	7.459
*gr666*	28.793	33.891	37.807	40.520

**Table 12:** Comparison of results.

Instance	$N_{g}$	ACS	PACO	DPSO-Ho		DPSO-He		ACO-SA	
		$C_{avg\%}$	$C_{avg\%}$	$C_{avg\%}$	$\sigma_{\%}$	$C_{avg\%}$	$\sigma_{\%}$	$C_{avg\%}$	$\sigma_{\%}$
*berlin52*	104	0.96%	0.96%	0.15%	0.32%	0.13%	0.15%	0.06%	0.12%
	416	0.50%	0.50%	0.01%	0.04%	0.01%	0.05%	0.01%	0.00%
	1664	0.46%	0.46%	0.00%	0.00%	0.01%	0.05%	0.00%	0.00%
*kroA100*	100	1.80%	2.97%	5.44%	2.47%	2.68%	1.40%	0.74%	0.24%
	400	1.31%	2.13%	1.28%	1.02%	1.05%	0.81%	0.29%	0.16%
	1600	0.82%	1.36%	0.64%	0.69%	0.78%	0.77%	0.17%	0.09%
*kroA200*	160	2.41%	3.33%	15.63%	2.77%	5.14%	1.84%	1.19%	0.35%
	640	1.62%	2.71%	4.45%	1.62%	2.89%	1.09%	0.81%	0.37%
	2560	1.47%	2.28%	1.62%	0.81%	2.02%	0.80%	0.63%	0.23%
*gr202*	128	6.26%	4.91%	13.75%	2.06%	4.19%	1.20%	2.21%	0.77%
	512	4.88%	3.90%	6.81%	2.11%	1.97%	0.66%	1.34%	0.56%
	2048	3.93%	3.34%	1.52%	0.60%	1.53%	0.55%	0.97%	0.44%
*pcb442*	272	6.18%	4.44%	29.31%	5.33%	6.73%	1.68%	3.07%	0.44%
	1088	4.87%	3.56%	13.41%	5.00%	2.87%	0.89%	2.83%	0.32%
	4352	3.91%	3.30%	3.13%	1.52%	1.92%	0.79%	2.56%	0.30%
*gr666*	384	9.18%	5.89%	10.84%	1.52%	9.58%	0.86%	4.46%	0.98%
	768	7.46%	4.77%	7.37%	1.00%	6.88%	0.78%	3.57%	0.70%
	1536	6.09%	4.51%	5.62%	0.84%	5.33%	0.57%	3.49%	0.65%
	3072	5.67%	4.14%	4.88%	0.63%	4.52%	0.88%	3.22%	0.66%
	6144	4.92%	4.21%	3.99%	0.77%	3.80%	0.78%	3.06%	0.51%
**Average values**		3.74%	3.18%	6.49%	1.56%	3.20%	0.83%	1.73%	0.39%

**Table 13:** Comparison of optimization times.

Instance	$N_{g}$	Optimization Time T (s)		
		DPSO-Ho	DPSO-He	ACO-SA
*berlin52*	104	0.13	0.13	0.06
	416	0.30	0.28	0.22
	1664	0.98	0.89	0.90
*kroA100*	100	1.03	0.86	0.27
	400	1.63	1.27	1.10
	1600	4.11	3.38	4.51
*kroA200*	160	2.49	2.18	2.02
	640	5.13	4.46	7.81
	2560	15.60	13.18	32.02
*gr202*	128	8.82	8.17	1.78
	512	11.54	10.88	7.07
	2048	23.01	21.98	28.53
*pcb442*	272	11.22	11.16	19.63
	1088	28.52	30.69	81.39
	4352	102.78	108.25	312.59
*gr666*	384	85.19	91.83	58.95
	768	98.36	115.19	116.42
	1536	124.84	163.48	241.68
	3072	180.66	259.00	460.09
	6144	296.83	453.83	951.12
**Average values**		50.16	65.05	116.41
